# Impact of Health Information Exchange on Emergency Medicine Clinical Decision Making

**DOI:** 10.5811/westjem.2015.9.28088

**Published:** 2015-12-14

**Authors:** Bradley D. Gordon, Kyle Bernard, Josh Salzman, Robin R. Whitebird

**Affiliations:** * HealthPartners Institute for Education and Research, Bloomington, Minnesota; † University of Minnesota Medical School, Academic Health Center, Minneapolis, Minnesota; ‡ Advocate Christ Medical Center, Chicago, Illinois; § Critical Care Research Center, Regions Hospital, Saint Paul, Minnesota; ¶ University of St. Thomas, School of Social Work, Saint Paul, Minnesota

## Abstract

**Introduction:**

The objective of the study was to understand the immediate utility of health information exchange (HIE) on emergency department (ED) providers by interviewing them shortly after the information was retrieved. Prior studies of physician perceptions regarding HIE have only been performed outside of the care environment.

**Methods:**

Trained research assistants interviewed resident physicians, physician assistants and attending physicians using a semi-structured questionnaire within two hours of making a HIE request. The responses were recorded, then transcribed for qualitative analysis. The transcribed interviews were analyzed for emerging qualitative themes.

**Results:**

We analyzed 40 interviews obtained from 29 providers. Primary qualitative themes discovered included the following: drivers for requests for outside information; the importance of unexpected information; historical lab values as reference points; providing context when determining whether to admit or discharge a patient; the importance of information in refining disposition; improved confidence of provider; and changes in decisions for diagnostic imaging.

**Conclusion:**

ED providers are driven to use HIE when they’re missing a known piece of information. This study finds two additional impacts not previously reported. First, providers sometimes find additional unanticipated useful information, supporting a workflow that lowers the threshold to request external information. Second, providers sometimes report utility when no changes to their existing plan are made as their confidence is increased based on external records. Our findings are concordant with previous studies in finding exchanged information is useful to provide context for interpreting lab results, making admission decisions, and prevents repeat diagnostic imaging.

## INTRODUCTION

### Background

The use of electronic health information exchange (HIE) offers the hope of increased provider efficiency, decreased diagnostics utilization and decreased administrative costs.^1^–^3^ The emergency department (ED) is a primary target for improvement, where providers make decisions on high volumes of unfamiliar patients in the absence of prior information.^4^–^6^

Studies and provider perception indicate cost savings can occur when HIE is used,^1^–^3^,^7^–^12^ such as in the decreased use of diagnostic imaging.^13^–^15^ Previous qualitative studies were conducted with the provider during non-clinical time,^16^–^19^ with two others adding clinical workflow observation to their interview methodology.^16^–^20^

### Importance

Determining how ED providers integrate HIE information can be logically expected to increase the value and decrease barriers to use, resulting in routine adoption to maximize the benefits for our care system overall.

### Goals of this Investigation

This mixed-method pursued the nuanced utility of HIE technology on providers’ clinical decisions by collecting the specific reasons for making an information request and the specific utility of the information retrieved during an individual patient encounter.

## METHODS

### Study Design

This prospective observational mixed-methods study used a brief semi-structured provider interview performed by a research assistant. This was completed within two hours of electronically requesting external records via HIE technology. The recorded interviews were transcribed for subsequent analysis. The institutional review board approved the study protocol.

### Study Setting

This study was performed in a single urban tertiary care hospital staffed with board-certified emergency medicine physicians, residents and physician assistants in a Midwestern state between June and August of 2013. The institution has used an integrated electronic health record (EHR) in use since 2006 (Epic Systems Corporation, Verona, WI). The vendor-supplied HIE technology has been in use since 2011 (Epic CareEverywhere). All providers are trained and experienced at viewing records through this HIE system and no additional login steps are required. At the time of the study, the hospital could request and receive records from over 70% of the regional acute care hospitals and 50% of the ambulatory clinics. This high regional density results in a high proportion of requests, resulting in detailed data.

### Study Participants

Interviews were conducted with the primary emergency medicine provider for an ED patient encounter, often a resident or physician assistant. Medical students and the principal investigator were not interviewed.

Enrollments resulted from a convenience sample of patient encounters during hours of research assistant coverage (1200 to 2300 daily). The ED clerk notified the research assistant of a new request for external records to generate candidates.

The study goals and methods were announced via meetings and email. Providers were excluded if they chose to opt out of the entire study at any point, or could decline an individual interview for any reason, without opting out of future enrollments. Interviews were limited to a maximum of two encounters per provider to limit bias.

### Methods of Measurement

The interview was conducted using a digital recorder and computer using an interview script embedded within a secure web-based data capture system. Categorical questions were captured as discrete responses and all other interview content was transcribed for subsequent qualitative analysis. The interview contained a total of seven questions, four of which had open-ended qualitative components. The investigators planned an interim analysis after 40 interviews had been performed and coded, based on prior experience. If that analysis determined that thematic saturation were reached, no additional enrollments would be performed.

### Primary Data Analysis

The type of clinical information providers sought and obtained were categorized from the interview transcripts. We used a content analysis approach to identify emerging themes and constructs from interview transcripts. Content analysis is an iterative process that uses a constant comparative method.^21^

## RESULTS

During the two-month study period, we obtained 40 interviews from 29 providers. Of the 29 providers, seven were attendings, 11 were residents and 11 were physician assistants. No providers prospectively opted out from the study or declined an interview request.

Of the 40 encounters studied, 93% (37) resulted in successful retrieval of electronic records from an outside institution. In the three failed requests providers did not fall back to requesting records via fax machine, which is the only other option to retrieve external records on patients where the CareEverywhere connection was not successful.

Of the 37 successful cases, providers reported a change in clinical decision-making in 32% (12) of the encounters and no change in 66% (25) of the encounters. In three of the patients where no change was made, providers reported increased confidence in their existing management plan after obtaining additional information. Ninety-two percent (34) of cases had a specific information need in mind when making a request, but in 38% (14) pf cases, unanticipated useful information was retrieved.

### Qualitative Themes

Corresponding example quotes for each theme are presented in the Table.

#### Specific Information Needs are Driving Requests for Outside Information

Providers initiated requests when specific information needs existed, most often prior test results and visit notes. In some cases, providers were searching for a broader target, such as lists of prior diagnoses or medications.

#### The Importance of Unexpected Information

Providers reported finding helpful unexpected information. Often they learned of recent visits for similar concerns at other healthcare settings.

#### Increased Confidence in Decision Making

Some providers noted that they didn’t make a change to their management plan, but found the external information increased their confidence in their existing management plan.

#### The Importance of Information in Disposition Decisions

Providers reported external information provided better context for making a disposition decision.

#### Prior Lab Results Serve as Important Reference Points

Prior lab results were also identified as important information needs, particularly to assist interpretation of lab results obtained during the current encounter.

#### Changes in Decisions for Diagnostic Imaging

When prior imaging results were available, providers often changed their plan to prevent repeat diagnostic imaging studies.

## DISCUSSION

This small-sample, mixed-methods survey is primarily hypothesis generating, and our discussion is focused on the nuances not found in previously reported work on HIE.

Providers in our study were driven to request records only when they had a specific need, an expected finding. Often the need was very specific, such as a specific test result. Less frequently, the need was general, such as a diagnosis or medication list. Unertl et al. similarly identified that providers commonly use HIE when prompted by learning of a recent encounter at another hospital.^20^

However, in one third of encounters, providers found useful unexpected information. Therefore, a measurable amount of helpful data exists but providers don’t know to ask for it. Automated requests to the HIE are routine aspects of some systems.^2^ Technology, policy and workflow changes designed to routinely trigger HIE requests may further enhance the known HIE utility and benefits.

A small number of providers who reported no change to their plan as a result of HIE information reported increased confidence based on the information, a finding not reported in previous studies. Further study would be needed to determine the impact of this increased confidence on provider and patient satisfaction and other outcomes.

Providers identified the importance of historical lab values as a key aspect of HIE. This finding may have implications for user interface design. As our health record system does not integrate external and internal results into the same view, future system design changes may find reduced barriers to HIE data use through safely co-mingling external data with internal data to providers with a more streamlined method of placing external data into the proper context.

When deciding whether to admit or discharge at the conclusion of an ED visit, providers report that HIE was valuable in providing context for a specific patient. This is concordant with other studies identifying potential reductions in admissions if HIE is used.^7^,^8^ It has been stated that deciding to admit someone to the hospital “may be the most expensive, regular discretionary decision in U.S. healthcare.”^22^ Providing improved awareness of a patient’s history may help target the use of expensive hospital beds to those who appropriately need these resources. It may be helpful to routinely collect external information on patients for whom the decision to admit or discharge is not a clear-cut one.

Further study is warranted to identify characteristics of patients that indicate an unknown information gap exists. For example, patients who report taking medications that aren’t on file locally may be likely to have detailed care information elsewhere. Even small markers of external information may help provide important context to a provider who is trying to create the best plan of care. Until an ED has eliminated all workflow barriers to routine HIE in all patients, our study seems to indicate providers will only jump through the hoops of HIE when they know there is something out there they need. Realistically, many organizations are not even close to routine and seamless HIE, so further study may help define which patients have a better outcome for their presenting problem when the provider has all the context needed for that patient’s care.

## LIMITATIONS

Only one quarter of providers interviewed were attending physicians and represents only one hospital’s ED experience, both of which may limit the generalizability of our findings. The convenience sample under-represents the experiences of providers who do not make HIE requests and excludes late night and early morning hours when there are fewer options of obtaining health information. Clinical decisions later in a patient encounter but after the provider interview may under-represent the impact of HIE.

## CONCLUSION

We found that providers report that information collected via electronic exchange was the direct cause of a change in clinical decision making one third of the time. Providers usually have a key piece of information in mind when requesting external records, but often find unanticipated information that they report as useful. Some instances of HIE use did not directly change decisions but the data were considered useful as it increased provider confidence in their plan. Themes emerged that may help guide workflow and software development in the domain of HIE.

## Figures and Tables

**Table:** Representative interview quotes of emergency physicians participating in study on health information exchange.

Themes identified	Representative interview quote
Specific information needs driving requests for outside information	I wanted her most recent echocardiogram report from her most recent cardiology visit and her most recent ED visit, if any, and I found the first two, and it doesn’t look like she had any recent ED visit. We had a woman who had problems with chronic abdominal pain and she wasn’t sure of what her actual diagnosis was and what workup had been done. But she was able to say she had been admitted … What I had wanted to get was just the discharge summary talking about what the diagnosis and what her previous workup was. Um, well specifically I was looking for medications [and] diagnoses, because I guess if he was on Coumadin I would have … umm, I don’t know if I would’ve changed [my plan].
The importance of unexpected information	[We found] that his Depakote was not specifically for seizures but for other psychiatric concerns … [it] would’ve otherwise … led us down more of a seizure pathway, as compared to ruling out a prior history of seizures. He was seen for a similar complaint two days prior. Yeah, he had frequent visits to the ED requesting uh, admission for both medical and behavioral health reasons. Um, with, uh, they felt a secondary gain, um, as motivation.
Prior lab results serve as important reference points	We did [make changes] because we found out that he had a baseline hemoglobin of like 7 to 8, so we held off on doing a transfusion. [We requested information on] baseline labs to see if there were any changes.
The importance of information in disposition decisions	[We] reviewed prior lab testing including a BMP, specifically looking at the patient’s sodium level … [The patient] will now be admitted under observation compared to being discharged home, after reviewing these tests.
Increased confidence in decision making	I think that just by knowing what her official diagnosis was and that the appropriate workup had been done, it was that reassurance that I didn’t do extra imaging … I am not sure that I would have done the imaging anyway, but it was reassuring and helpful. No, but it gave me some good background information and baseline labs and her appropriate medications that she is going to be on.
Changes in decisions for diagnostic Imaging	Because labs and imaging were done less than twenty-four hours ago at a different hospital, I did not do any additional testing that I would have done had we not been able to access the records. The patient is here and is about 5 weeks pregnant, and the question was whether or not she already had an ultrasound done elsewhere and actually she’s done them at three other hospitals and had ultrasounds done at all of those hospitals so I will not be doing an ultrasound here today.

*ED,* emergency department; *BMP,* basic metabolic panel
